# Concluding Commentary. The Importance of the Humanities in Medical Education: Where are we now?

**DOI:** 10.15694/mep.2018.0000220.1

**Published:** 2018-09-24

**Authors:** Jonathan McFarland, Irina Markovina, Trevor Gibbs

**Affiliations:** 1 Sechenov University; 2 AMEE

**Keywords:** Medical Humanities, MedEdPublish Theme Issue

## Abstract

This article was migrated. The article was marked as recommended.

The undergraduate medical curriculum, together with many of the other healthcare curricula, is under a constant state of change. Sometimes that change is for the better, very occasionally less so. Many physicians who graduated more than forty years ago may agree that the humanities were a strong component of the hidden curriculum; the humanities were just there and enhanced many teaching activities (mainly lectures) to a variable state. They were used by a certain proportion of faculty to make their lectures “more exciting”, “more real” and “to put them in context”. Over time and as new teaching and learning technologies took the place of formal teaching (such as lectures), these humanities approaches and enhancements appeared to become less prominent.

This new AMEE MedEdPublish theme - The Importance of the Humanities in Medical Education - has not only demonstrated that the humanities are not gone and lost forever, they were just hidden, but the number of papers received demonstrated that the subject is certainly healthier than expected and suggests the inclusion of the Humanities within all forms of healthcare curricula and training opportunities.

This concluding commentary provides an overview of the large number of papers received, explores the various reasons that support the presence of the humanities in the curriculum, provides insight into the ways that the humanities are being taught and finally, provides some direction of the way forward.

## Introduction

The opening editorial of this themed issue on The Importance of the Humanities in Medical Education concluded appropriately with the words of Sir William Osler, who, nearly one hundred years ago commented: *“Science and Humanities ... are twin berries on one stem, grievous damage has been done to both in regarding (them)..in any other light than complemental”.* ( Osler, 1919)

Furthermore, it was highlighted that even though it is not a new topic it still elicits much debate. There is still debate as to what constitutes the Medical Humanities, as to its role in medicine and medical education, or whether it is a “soft skill” which can be added to the hard currency of science. The articles received by a wide body of persons interested in this theme convincingly demonstrate the variety of approaches, multitudes of opinions and diversity of experiences; excellent ground for an interesting and enthusiastic debate.

In the opening editorial, the authors laid down the gauntlet, asking all those with an interest to send us their articles and open the debate on the *“What and Why and When / And How and Where and Who”* of the medical humanities. ( Kipling, 1942). The authors asked for ideas to flow and the debate to begin, ending their editorial with these words:

*“We look forward to reading your thoughts on this subject and we accept all forms of publications: research and descriptive papers, opinion pieces and personal views”.*

Perhaps before providing an overview of the papers and their subject matter, it is appropriate to thank all those who contributed so well to the theme; the response has far exceeded our expectations, with papers showing a broad diversity of subject matter. The articles published are illustrative of the present position of the humanities in medicine, with the great majority being personal views or opinions. Less numerous were the research papers, new educational technologies, reviews and practical tips. This spread of submissions is probably indicative of the humanities still being seen as a slightly subjective field. Also, of interest is the fact that the papers showed a diverse geographical spread, coming from eleven different countries. Despite a western preponderance, those papers coming from Asia, and the Caribbean demonstrate a very positive sign of the internationalisation of the medical humanities.

The format of AMEE MedEdPublish is a splendid way to promote debate within the medical education community, and this issue has been no exception. There has been a constant flow of new ideas and suggestions after the publication of the papers, which we hope will continue after the theme has closed. So, taking Kipling as a start, and taking into consideration that some articles overlap sub-themes, we aim to summarise the papers under the headings of WHY, WHERE, WHAT and HOW.

## WHY

The majority of the papers were published, probably as expected, under the heading of Personal views or opinions. Many of them demonstrated the passion that the authors hold for the humanities. Liao ( Liao, and Rumsey, 2018) emphasises the need to embed the humanities into the daily culture and teaching of medicine, focusing on the need to learn from the mistakes of the past. Their likening to Nazi eponyms may not feel comfortable for all, but as the authors state, many eponyms were applied from doctors with less than acceptable morals; a point of discussion in any humanities programme. Pera and colleague, ( Pera and Pera, 2018), two surgeons, one practising and the other retired, movingly and pertinently brought to the attention of all that dignity should always be placed at the forefront of the Doctor-patient relationship, highlighting the difference between illness and disease and Foucault’s “medical gaze”.

In Houston’s article ( Houston, 2018), entitled “ Medical Humanities: time to join the mainstream of medical education” he again highlights this critical issue. Although most of the reviews valued and agreed with the paper and could recognise Houston’s drive for the humanities, a large number of reviewers called for a need to move beyond the **why**, moving to the element of **how** do we place the humanities within the mainstream curriculum, a success in its own right.

Appan, ( Appan, 2018), a 4th-year psychiatry student studying in Singapore and also a classical Indian dancer contributed a very personal account of how dance had helped her restore her passion for her work, a slightly different effect of the humanities. It is a beautifully written piece advocating that medical students should be able to, *“... choose humanities of their interest, so as to incorporate it into their learning”.*

Zaharias ( Zaharias, 2018), an Australian General Practitioner brilliantly grapples with how to use Art in medical education, taking Luke Fildes’s painting The Doctor as an example, and asks the question *“is it not logical to turn to the arts (the humanities) for the lessons which science cannot teach?”*; perhaps a sentence that should be printed in bold and placed on the desks of all policymakers involved with medical education.

One of the most refreshing things emerging from this theme has been the strong input from practising doctors, those faced with treating patients on a daily basis; a good example comes from Dr Eduardo Morera Serna ( Morera Serna, 2018). His sharp, pertinent and passionate article calls for a return of humanities not only in medical education but also in all forms of education, for, as he says, *“science without a humanistic approach is useless and dangerous”.* Some strong words indeed, and much food for thought.

Other papers in this personal views section on how important the humanities are to medical education include Mostwin’s ( Mostwin, 2018) eloquently written piece on the life writing of eminent physicians (Korczak, Sacks, Schweitzer and Ishida, to name but a few), all of whom we can learn from, their stories shaping how we respond to patients and react to their stories. Mostwin quotes from Robert Lowell’s poem Epilogue, “ *Yet why not say what happened? Pray for the grace of accuracy..*” ( Lowell, 1977) Why not indeed, so that we can learn.

Dornan and Kelly (2018) change key and tone completely, bringing us their personal reflection on the relationship between music and medicine, for as they say, *“good doctors, like good musicians tune in to patients at a personal level”*, going on to state that “ *the Dr-patient .. union is founded on emotion.*” Lastly, in this section the paper by Stender and Stender (2018), explores one of their most important take-home messages; *“The art of and science of medicine are not dichotomous but synergistic”.*

## WHERE

One of the most rewarding aspects of this theme has been its internationality, and in this short section, we will look at some of the settings discussed in the papers.

The paper by Wu and Chen ( Wu and Chen, 2018) reviews the development of medical humanities pedagogies in Taiwan, China and Hong Kong and discusses the evolution of the humanities and the difficulties encountered across the three diverse societies. As the authors say *“It is time for developers of medical humanities, and more importantly, medical education at a higher level, to rethink and strategise the way that the teaching of medical humanities is positioned and delivered”.* They finish with this timely and pertinent warning - *“otherwise, the vision of medical humanities remains a castle in the air”.*

Lechopier and colleagues ( Lechopier *et al.,* 2018) explored the debate that took place in France in 2018 concerning the ethical and social issues of biomedicine and life science and technologies and how to “equip” tomorrow’s health professionals; those who will look after us when we are older, our children and perhaps our grandchildren? In their paper they list four crucial issues for the present and the future of healthcare profession: the changes of the roles people play in healthcare; new biomedical concepts and innovations; their long-term consequences on the health and social contract and the ethical issues in healthcare and daily life settings. They further describe four kinds of resources, including bringing together Social Sciences and Humanities courses that can hopefully resolve these crucial issues.

The papers do not miss out the importance of the student, and the paper by Soriano and his students ( Soriano et al., 2018) explores how important it is to have interests outside the specific medical curricula; indeed, they expound that this is essential to making the balanced and well-grounded professionals who will lead the medical profession into the future.

And lastly, in this section, Malpas and Jowsey (2018) draw on their 20 years of experience of teaching medical humanities at the University of Auckland through their establishment of a Student Selected Component. It is rich and powerful paper and should be read by all with an interest in this topic. They highlight how the vision central to pioneers of medical education such as William Osler, who recognised the importance and significance of the whole person and not just their illness, injury or disability, is now more critical than ever. Moreover, their poignant use of poetry written by their medical students movingly accentuates this.

## WHAT

Davin and colleagues’ paper entitled, “Compassion, the first emotion ditched when I’m busy’ ( Davin et al., 2018), asked first-year interns *“what have been the main influences (positive and/or negative) in how you have learned to express compassion for your patients when working in the clinical context?”* As they describe, *“the interns’ reflections uncovered a narrative of emotional vulnerability, where fearing failure and seeking perfection contributed to a diminished self‑efficacy, resulting in risk aversive behaviours protecting their doctor identity”.* According to the authors, this paper draws on the issues highlighted in the original opening editorial published on 9th July 2018, in which one of the reviewers commented on the “tensions between competence and caring”. The power of this paper can be seen in its last Take Home Message: *Do not allow attempting to be the perfect doctor eclipse being a kind and caring human being.* This paper has also been one of the most successful papers in terms of reviews and responses to reviews, and we wish to also disclose that after publication the author commented in a mail to the Theme Editor that “Whilst it makes one feel very vulnerable having your work out there for reviews, it’s been a very rewarding process”. ^*^Surely this is a true vindication of the merits of the post-publication review process; true democracy at work in scientific publishing.

Ledger and Joynes (2018) in their fascinating article explore the place of music in the development of future doctors, through the lens of a mixed method, longitudinal evaluation of a two-week music and medicine special studies project for second- and third-year medical students. Such a course would seem to be essential for allowing students to develop their own creativity, and it could also be seen in the light of a paper discussed previously ( Soriano et al., 2018) whereby stimulating medical students’ external interests could help them to develop their identities as doctors, and also help them maintain the passion needed to prosper as future professionals ( Appan, 2018).

## HOW

Tellingly this last section is also probably the key to whether we all succeed in our aim of restoring the humanities to its rightful place at the core of medical education. Below is listed some papers that have given concrete examples of how the humanities can be placed within the curriculum. Two early articles published were by Margaret Chisolm and her colleagues and explored the practical side of humanities teaching. The first paper ( Gelgoot et al., 2018) was a review of the literature to identify curricula that incorporate the visual arts into undergraduate, graduate, and continuing medical education to facilitate the teaching of clinical excellence. As well as providing an excellent list of useful references, the review supported the use of visual arts in medical education to facilitate the teaching of clinical excellence. Unfortunately, the review also uncovered the common issue, common to many papers related to the humanities, and specifically that of not providing data that evaluates the impact of the visual arts on clinical excellence outcomes.

The second article ( Chisolm et al., 2018) is a simple and fascinating idea of how to promote the reading of non-medical books by medical professionals, through the use of a book-club. A true reflection of the ideals of Osler, and moreover the paper shows that lay literature can play an important role in the development of clinicians, something which is still debated and truly underestimated.

The last two papers ( Hagazi and Hough, 2018) and ( Wald and Weiss, 2018) may offer similarities in their use of “real” experience in healthcare training; with the first paper being a thoughtful piece on patient-centredness and in making sure that the “real” is truly introduced into Problem Based Learning. The second is an interesting “exemplar” of how reflective writing can provide “real” and useful feedback for trainees. In the conclusion, Wald and Weiss accentuate the “power of the pen”, stating that using both professional and personal experience “ *may even make the feedback process “more real” to support one’s development as a reflective, resilient and humanistic healthcare professional*”.

## Conclusion

We appreciate that in this concluding commentary we have not discussed all the papers published. That is not a reflection of the standard of the papers, more a reflection of the summarising process. They were all useful to read and all drew out some interesting aspects of the teaching of the humanities in medical education. It is also equally important to underline that this is an on-going story; a short introduction to a thorough, detailed and multidisciplinary discussion of one of the most important problems facing modern medical education and the profession, in general how to introduce or reintroduce the humanities. We hope and presume that the promising debate initiated in this issue will continue. As one author has stated - *“Humanistic education helps to cope with the ups and downs of daily clinical work building resilient physicians. Investing in medical humanities to achieve top professionals may be one of the soundest ideas of medical schools”* ( Morera Serna, 2018)

It is our aim that, by using this themed issue as a base, we can help ensure that the medical humanities do not remain a “castle in the air”. ( Wu and Chen, 2018) The stakes are too high. The future of medicine depends on finding the way to (re)introduce the humanities to the core of medical education, and the future generations depend on this, but as is so often the case, “ *Time present and time past/ Are both perhaps present in time future/ And time future contained in time past*” ( Eliot T.S, 1970).

Never have these words been truer:

*“Wherever the art of medicine is loved, there is also a love of humanity”.* ( Hippocrates, 2018)

## Take Home Messages

We believe that the following could be seen as what we have learned from this themed issue:

• **There appears to be a lack of a consistent definition of the Medical Humanities:**
  • different interpretations and a great diversity of interpretations
  • differences between medical or healthcare education
• **Wide experience has been accumulated and continues to be accumulated in the (the field of) teaching the Medical Humanities**:
  • broad variety of activities and projects
  • many novel and innovative teaching methods
• **The way forward to find the place Humanities in Medical Education:**
  • as society changes, healthcare education needs to change to reflect the differences in society and the need for more humanism
  • integrated approaches to Humanities teaching are preferable to stand-alone courses, with interprofessionality being a critical part of the humanities curriculum
  • short and long-term evaluation of the effects of teaching the Humanities on our future healthcare practitioners is key to its acceptance as a permanent feature of healthcare curricula

## Notes On Contributors

**Mr. Jonathan McFarland** is the Head of Academic Writing at Sechenov First State Medical University in Moscow, and a member of their international faculty. He currently holds the position of President of The Doctor as a Humanist Association, a new Association, which held its first international symposium in October 2017, and which aims to promote and develop internationally the concept of the humanities within undergraduate and postgraduate healthcare education.

**Professor Irina Markovina** is Director of Institute of Linguistics and Intercultural Communication at Sechenov University (Sechenov First Moscow State Medical University). Her interests lie in Psycholinguistics, and she is a representative of the Russian School of Psycholinguistics as well as an Editorial Board member of the peer-reviewed Russian journal “Problems of Psycholinguistics”. She is also committed to developing the English language environment at Sechenov and in other medical universities.

**Professor Trevor Gibbs** is an Independent Consultant in Medical Education and Primary Care development. He is also AMEE International Development Officer and Associate Editor of AMEE MedEdPublish. His interests lie in curriculum transformation and development, the Social Accountability of medical schools and teaching, learning and assessment methodologies.

## Declarations

Mr Jonathan McFarland and Professors Trevor Gibbs and Irina Markovina are AMEE MedEdPublish Theme Editors.Professor Trevor Gibbs is also an Associate Editor of AMEE MedEdPublish.
