# Supplementary material for: Concluding Commentary. The Importance of the Humanities in Medical Education: Where are we now?
Source: MedEdPublish (2016). 2018 Sep 24;7:220. [Version 1] doi: 10.15694/mep.2018.0000220.1 (PMC10704416; doi:10.15694/mep.2018.0000220.1)
Supplement: Peer review history

# Reviewer report: Reviewer response for version 1

**DOI:** 10.21956/mep.19676.r29388

**Published:** 2018-10-08

**Authors:** Barbara Jennings

**Affiliations:** 1 Norwich Medical School

This review has been migrated. The reviewer awarded 5 stars out of 5 What a successful and stimulating themed edition of MedEdPublish this has been. I teach genetics and molecular biology, but some of the papers linked to this humanities-themed-issue (coupled with some creative presentations from the AMEE2018 fringe, by students using the performing arts) are probably going to have the biggest influence on me as a teacher in the academic year ahead. The Why, Where, What & How structure of this closing editorial neatly allowed the authors to consider the heart-felt and logical rationale for bringing arts and humanities into our medical education classrooms and to cite and summarise many of the papers that comprised this issue. The authors were also able to report and reflect on the challenges and opportunities that post-publication-peer-review added to this global conversation about diverse interventions; including visual arts, music, and poetry.The cited studies presented many sound reasons, and good evidence for integrating the humanities within a medical education; and the authors of this editorial highlight the need for more research and studies of outcomes and measurable impact (on students, on patients, and on educators) to firmly secure that position.

Reviewer Expertise:

NA

No decision status is available

# Reviewer report: Reviewer response for version 1

**DOI:** 10.21956/mep.19676.r29387

**Published:** 2018-09-24

**Authors:** P Ravi Shankar

**Affiliations:** 1 American International Medical University

This review has been migrated. The reviewer awarded 5 stars out of 5 I enjoyed being a part of the reviewer panel for this special themed issue on the medical humanities. There has been a rich collection of articles on the medical/health humanities. The number of articles was much greater than my expectations and the post-publication peer review process and the authors’ response to the reviewers initiated a rich dialogue as mentioned by the editors. Hoping the medical humanities will continue to grow and spread globally and educators are able to use examples and concepts from within their own cultures and settings. Thank you for bringing out this special issue.

Reviewer Expertise:

NA

No decision status is available
